# Author Correction: c-di-AMP signaling plays important role in determining antibiotic tolerance phenotypes of *Mycobacterium smegmatis*

**DOI:** 10.1038/s41598-022-20905-1

**Published:** 2022-09-28

**Authors:** Aditya Kumar Pal, Anirban Ghosh

**Affiliations:** Molecular Biophysics Unit, Indian Institute of Science, Bangalore, 560012 India

Correction to: *Scientific Reports* 10.1038/s41598-022-17051-z, published online 30 July 2022

The original version of this Article contained errors in the legend of Figure 4B, the Supplementary Figure S4 and in the Results section.

In the legend of Figure 4B,

“(**B**) Slower resuscitation of *Δpde* persister cells results in a rapid enrichment of genetic mutants, around 93% of mutant population enrichment happened in *Δpde* strain within 24 h of the regrowth phase, whereas in the case of WT strain ~ 52% of cells became ciprofloxacin mutants [N = 3].”

now reads:

“(**B**) Slower resuscitation of *Δpde* surviving cells results from a rapid enrichment of genetic mutants, around 93% of mutant population enrichment happened in *Δpde* strain within 24 hours of the killing, whereas in the case of WT strain ~ 52% of cells became ciprofloxacin mutants [N = 3].”

In the legend of Supplementary Figure S4,

“Regrowth of persisters **(a)** in presence of a low concentration of ciprofloxacin (3X MIC) for WT and *Δpde* strains and **(b)** in the complete absence of ciprofloxacin (10X treatment followed by washing off the drug) for *Δpde and Δpde disA-fs* strains.”

now reads:

“Regrowth of surviving cells **(a)** in presence of a low concentration of ciprofloxacin (3X MIC) for WT and *Δpde* strains and **(b)** in the complete absence of ciprofloxacin (10X treatment followed by washing off the drug) for *Δpde and Δpde disA-fs* strains.”

In addition, in the Results section, under the subheading ‘c-di-AMP plays a role in persister cells regrowth by modulating resuscitation-promoting factor gene *rpfA* expression,”

“Next, we checked if the slower resuscitation of *Δpde* persister cells could result in more genetic mutants, we estimated the number of ciprofloxacin-resistant genetic mutants (cip^R^) by plating parallelly on ciprofloxacin containing plate. We found a visible increase in cip^R^ mutants in the case of *Δpde* mutant strain which attained a remarkable ~ 93% mutant population takeover within 24 h of the regrowth phase, whereas in the case of WT strain it was ~ 52% of cells became ciprofloxacin mutants (Fig. 4B).”

now reads:

“Next, we checked if the slower resuscitation of *Δpde* persister cells could result from more genetic mutants, we estimated the number of ciprofloxacin-resistant genetic mutants (cip^R^) by plating parallelly on ciprofloxacin containing plate. We found a visible increase in cip^R^ mutants in the case of *Δpde* mutant strain which attained a remarkable ~ 93% mutant population takeover within 24 h of the killing, whereas in the case of WT strain it was ~ 52% of cells became ciprofloxacin mutants (Fig. 4B).”

The original Article and the error in the Supplementary Information file have been corrected.

## Supplementary Information

Supplementary Information.

