# Supplementary material for: c-di-AMP signaling plays important role in determining antibiotic tolerance phenotypes of Mycobacterium smegmatis
Source: Sci Rep. 2022 Jul 30;12:13127. doi: 10.1038/s41598-022-17051-z (PMC9338955; doi:10.1038/s41598-022-17051-z)
Supplement: Supplementary file 1 — Supplementary Information. [file 41598_2022_17051_MOESM1_ESM.pdf]

Figure S1

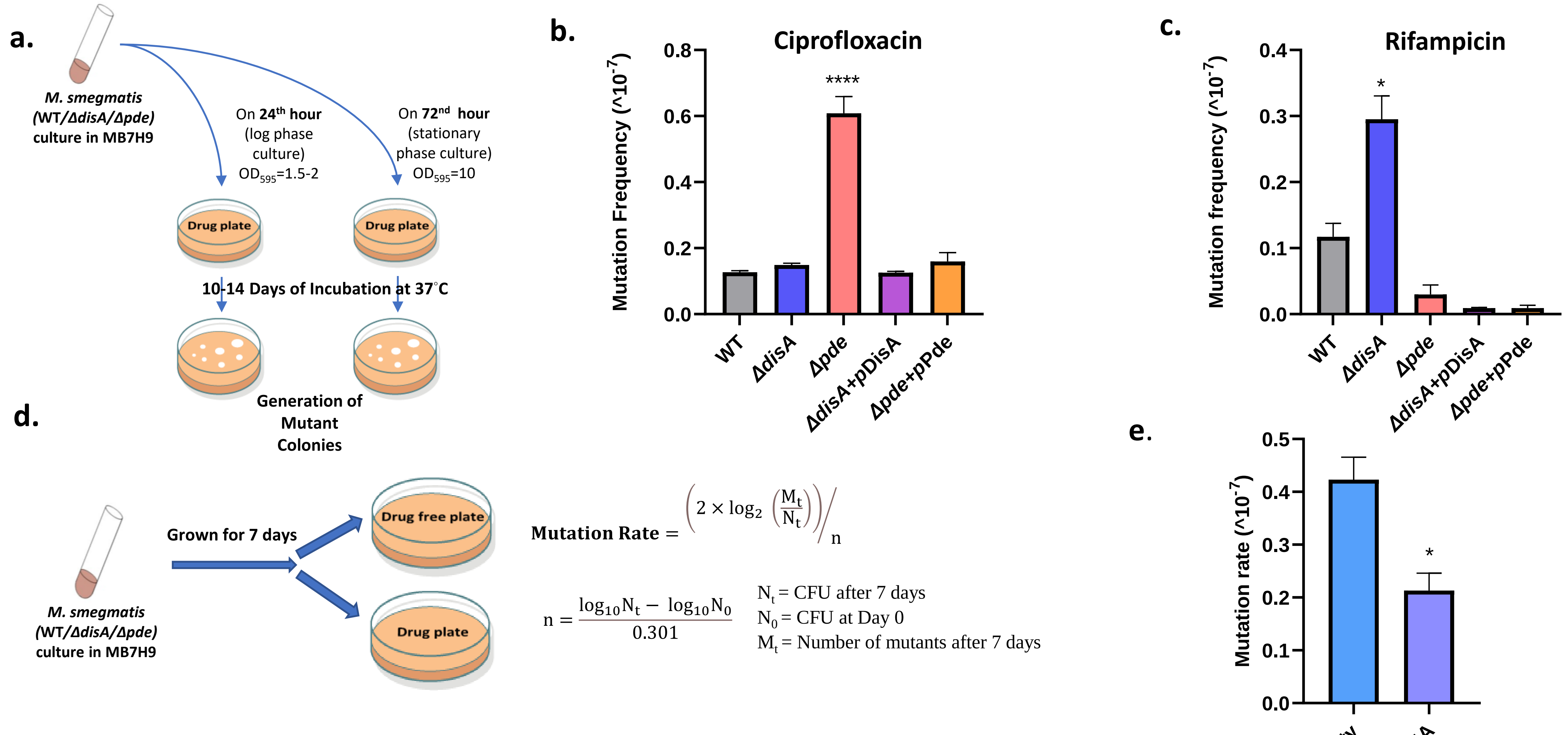

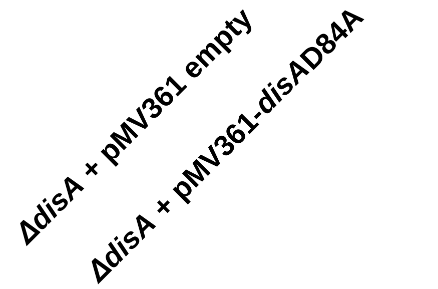

Figure S1: **(a)** Schematic illustration of the experimental setup to enumerate mutation frequency; Mutation frequency determination using exponentially grown cultures of the respective strains for **(b)** ciprofloxacin and **(c)** rifampicin [N=3]. **(d)** Schematic illustration of the experimental setup to enumerate mutation rate along with the calculation formula; **(e)** Mutation rate (rifampicin) calculation for *ΔdisA* strain when complemented with either pMV361-Empty or pMV361-*disA(D84A)* plasmid.

Figure S2

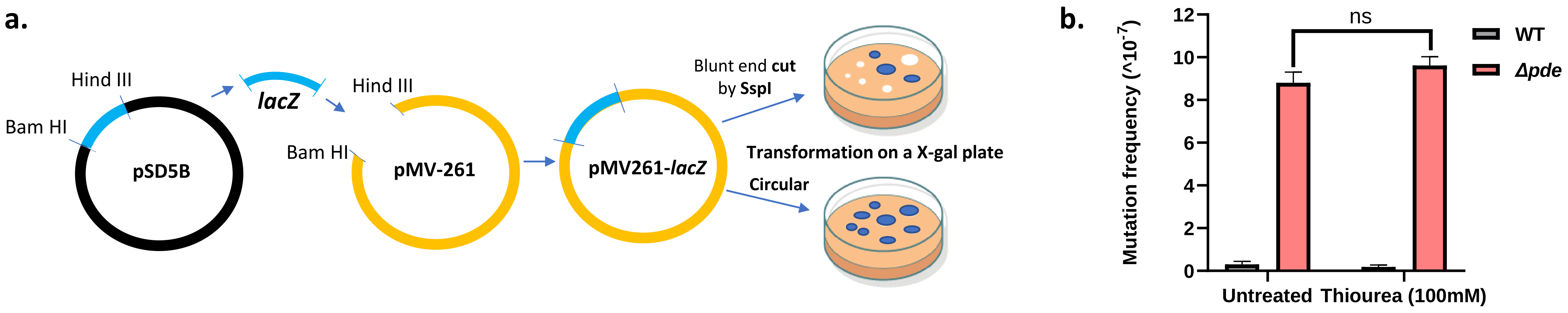

Figure S2: **(a)** Schematic representation depicting the construction of the *lacZ*-reporter plasmid and NHEJ-driven plasmid repair and fidelity assay; **(b)** Ciprofloxacin mutation frequency determination of the respective strains in the presence and absence of thiourea (100mM).

Figure S3

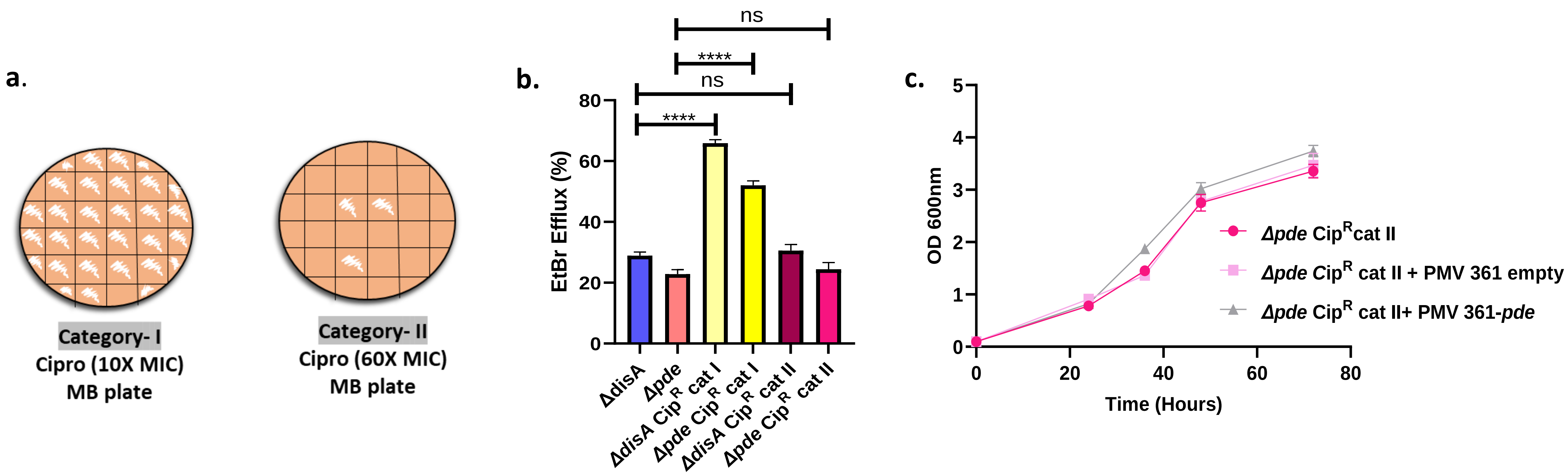

Figure S3: (a) Schematic illustration of the screening for category-I and category-II cipR mutants; **(b)** EtBr efflux assay for different categories of *M. smegmatis ΔdisA* and *M. smegmatis Δpde* cipR mutants; **(c)** Minimal media growth curve depicting fitness cost of respective mutant strains.

Figure S4

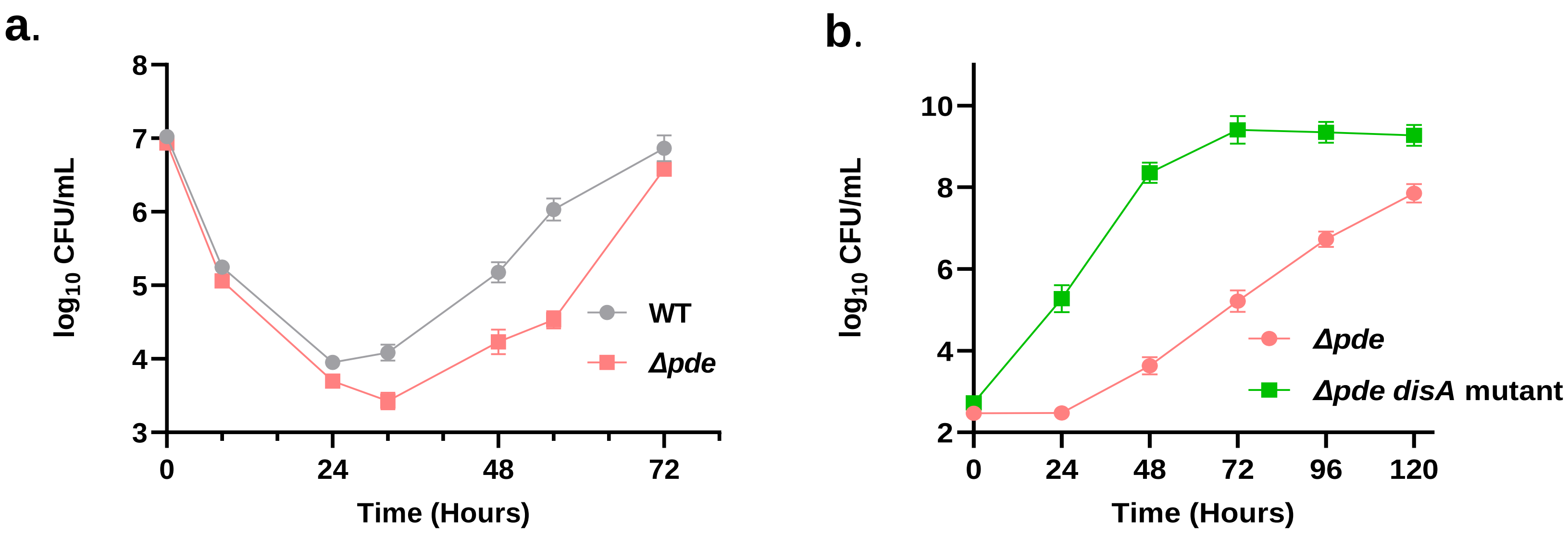

Figure S4: Regrowth of surviving cells **(a)** in presence of a low concentration of ciprofloxacin (3X MIC) for WT and *Δpde* strains and **(b)** in the complete absence of ciprofloxacin (10X treatment followed by washing off the drug) for *Δpde and Δpde disA-fs* strains.

Table S1: Sequencing confirmation of frameshift mutations at the repair sites of *Δpde* + pMV261-*lacZ* white clones

Sl No.	***Δpde LacZ* mutants**	**(SspI cut site - AATATT)**
**1**	**AATTATT**	T insertion
**2**	**AATTATT**	T insertion
**3**	**A--ATT**	AT deletion
**4**	**AATTATT**	T insertion
**5**	**AATAATT**	A insertion

Table S2: Ciprofloxacin MIC values of the respective strains

	**MIC (μg/ml)**
**Strain**	**Ciprofloxacin**
**WT + pMV261 empty**	0.25
***Δpde* + pMV261 empty**	0.125
**WT + pMV261 *ku*-AS**	0.25
***Δpde* + pMV261 *ku*-AS**	0.0625

Table S3: Ciprofloxacin MIC values of the parental and mutant strains

Strain	MIC (µg/ml)
	**Ciprofloxacin**
**WT**	0.25
**WT cip^R cat I**	8
**WT cip^R cat II**	64
ΔdisA	0.25
***ΔdisA* cip^R cat I**	8
***ΔdisA* cip^R cat II**	64
**Δpde**	0.125
***Δpde* cip^R cat I**	4
***Δpde* cip^R cat II**	32
**WT + CCCP (5µg/mL)**	0.03
**WT cip^R cat I + CCCP**	≤0.125
**WT cip^R cat II + CCCP**	16

Table S4: Sequencing confirmations of different amino acid substitutions and frameshifts in *gyrA* and *lfrR* genes of the cipR mutants

Sl No.	Strain	Mutant sequencing	
		**QRDR of *gyrA* (MSMEG_0006)**	***lfrR* (MSMEG_6223)**
1	**WT cip^R cat I**	No mutation	L59fs
2	**WT cip^R cat II**	D94Y (2)<br>D94G (2)	NA
3	***ΔdisA* cip^R cat I**	No mutation	H29fs
4	***ΔdisA* cip^R cat II**	D94G (2)<br>D94Y (1)	NA
5	***Δpde* cip^R cat I**	No mutation	P167fs
6	***Δpde* cip^R cat II**	D94N (1)	NA

Table S5: Rifampicin MIC values of the parental and mutant strains

Strain	MIC (µg/ml)
	**Rifampicin**
Δpde	2
***Δpde* Rif^{R} mutant**	512
***Δpde* II Cip^{R} Rif^{R} double mutant**	32

Table S6: Bacterial strains used in the study

Strains	Description	Source/ Reference
M. smegmatis Wild type (WT)	*M. smegmatis* mc^2 155	Laboratory stock
M. smegmatis Δ*disA*	*M. smegmatis* mc^2 155 strain knock out for disA ; Kan^R	Ref.1
M. smegmatis Δ*pde*	*M. smegmatis* mc^2 155 *strain knock out for pde ;* Kan^R	Ref.1
M. smegmatis Δ*disA* +*p*DisA	*M. smegmatis* Δ*disA* strain containing disA cloned in pMV361 vector; Hyg^R,Kan^R	Ref.1
M. smegmatis Δ*pde* +*p*Pde	*M. smegmatis* Δ*pde* strain containing pde cloned in pMV361 vector; Hyg^R,Kan^R	Ref.1
M. smegmatis Δ*disA* +*disA* D84A	*M. smegmatis* Δ*disA* strain containing disA (D84A) cloned in pMV361 vector; Hyg^R,Kan^R	Ref.1
M. smegmatis Δ*disA* + pMV361 empty	*M. smegmatis* Δ*disA* strain containing pMV361 empty vector; Hyg^R,Kan^R	Ref.1
M. smegmatis WT +pMV261 empty	*M. smegmatis* WT strain containing pMV261 empty vector; Hyg^R,Kan^R	This study
M. smegmatis Δ*pde* +pMV261 empty	*M. smegmatis* WT strain containing pMV261 empty vector; Hyg^R,Kan^R	This study
M. smegmatis WT + pMV261 *ku*-AS	*ku*-anti sense knockdown construct *(MSMEG_5580)* in pMV261:hyg vector transformed into *M. smegmatis* WT	This study
M. smegmatis Δ*pde* + pMV261 *ku*-AS	*ku*-anti sense knockdown construct *(MSMEG_5580)* in pMV261:hyg vector transformed into *M. smegmatis* Δ*pde*	This study

Table S6 (cont..)

Strains	Description	Source/ Reference
M. smegmatis Δpde disA mutant	*M. smegmatis Δpde* with a frameshift mutation in *disA* gene	This study
M. smegmatis WT cip^R cat I	Ciprofloxacin resistant mutant of *M. smegmatis* WT having mutation in *lfrR* gene	This study
M. smegmatis WT cip^R cat II	Ciprofloxacin resistant mutant of *M. smegmatis* WT having mutation in *gyrA* gene	This study
M. smegmatis ΔdisA cip^R cat I	Ciprofloxacin resistant mutant of *M. smegmatis ΔdisA* having mutation in *lfrR* gene	This study
M. smegmatis ΔdisA cip^R cat II	Ciprofloxacin resistant mutant of *M. smegmatis ΔdisA* having mutation in *gyrA* gene	This study
M. smegmatis Δpde cip^R cat I	Ciprofloxacin resistant mutant of *M. smegmatis Δpde* having mutation in *lfrR* gene	This study
M. smegmatis Δpde cip^R cat II	Ciprofloxacin resistant mutant of *M. smegmatis Δpde* having mutation in *gyrA* gene	This study
M. smegmatis Δpde cip^R cat II rif^R double mutant	Mutant of *M. smegmatis Δpde* having resistance to ciprofloxacin and rifampicin	This study
M. Smegmatis Δpde rif^R mutant	Rifampicin resistant mutant of *M. smegmatis Δpde* having mutation in *rpoB* gene	This study
WT cip^R cat II + pMV 361 empty	*M. smegmatis* WT Cip^R cat II having pMV361 vector; Hyg^R	This study
WT cip^R cat II + pMV 361 *pde*	*M. smegmatis* WT Cip^R cat II having pMV361 vector; Hyg^R	This study
M. smegmatis WT + *Prpfa*-GFP	*M. smegmatis* WT with promoter fusion construct (434us) rpfa- $pMN406_{\Delta imyc}$; Hyg^R	This study
M. smegmatis Δpde + *Prpfa*-GFP	*M. smegmatis Δpde* with promoter fusion construct (434us) rpfa- $pMN406_{\Delta imyc}$; Hyg^R	This study
M. smegmatis Δpde disA mutant + *Prpfa*-GFP	*M. smegmatis Δpde disA* mutant with promoter fusion construct (434us) *(rpfa-* $pMN406_{\Delta imyc}$ *;* Hyg^R	This study
Δ*pde* + pMV261 *lacZ*	*M. smegmatis Δpde* with *lacZ* gene cloned in pMV261 vector *;* Hyg^R	This study

Table S7: Primers used in the study

**Name**	**Sequence (5’ to 3’)**	**Description**
lacZ_PMV261FW_hindIII	AGCT*AAGCTT*TCCGGAGGAATCACTTCCAT	To construct the NHEJ reporter vector
lacZ_PMV261REV_hindIII	TCAG*AAGCTT*TTATTTTTGACACCAGACCA	To construct the NHEJ reporter vector
Ku_AS_REV_HindIII	ATCGAAGCTTATGAACCGTGCGGTACGCCA	To construct knock down of *ku* gene
Ku_AS_FW_BamHI	ACTAGGATCCCTACGACTTCTTCGCAGCTG	To construct knock down of *ku* gene
Ms_QRDR FW	GACCGACATCGGTGGGTTGC	To amplify QRDR region of *gyrA*
Ms_QRDR Rev	AATCGACTGTCTCCTCGTCG	To amplify QRDR region of *gyrA*
lfrR_fw	ACCGCCTCCGGCGGTGCCGGCT	To amplify QRDR region of *lfrR and also u*sed for sequencing of *lfrR*
lfrR_rev	GCCACCCAGGCGCGCGTGGGCG	To amplify QRDR region of *lfrR*
rrdr_fw	GTCGTCTGCGCACCGTCGG	To amplify RRDR region of *rpoB and also u*sed for sequencing of RRDR
rrdr_rev	CTCGATGAAGCCGAACGGG	To amplify RRDR region of *rpoB*
gyrA_fw	ATGACTGATACGACGCTGCCGC	Used for sequencing of *gyrA*
lacZ_mid_rev_2	GCCTTCATACTGCACCGGGCG	Used for sequencing of *lacZ*
disa_mid_rev	GGTCATCACGTCGCGCAGCG	Used for sequencing of *disA*
rpfa_434bpus _FW_Xba1	AGCAGTCTAGACGTGAGATACGTCACATC	Used for making *PrpfA*-GFP transcriptional fusion construct
rpfa_434bpus_Rev_Sph1	TCATAGCATGCAAGCGTCGAGGTCCTCTC	Used for making *PrpfA*-GFP transcriptional fusion construct

Supplementary References:

1. Chaudhary, V., Pal, A., Singla, M. & Ghosh, A. Elucidating the role of c-di-AMP in Mycobacterium smegmatis: phenotypic characterization and functional analysis. *bioRxiv* 2022.03.25.485789 (2022) doi:10.1101/2022.03.25.485789
